# The Adamson Collection: illustrations of mental illness or a testament to spontaneous artistic expression?

**DOI:** 10.3109/17453054.2015.1108297

**Published:** 2016-01-30

**Authors:** Anna Ostrowska

**Affiliations:** ^a^ Wellcome Library, London, UK

## Introduction

British artist Edward Adamson (1911–1996) was one of the pioneers of art therapy in the UK, working first in a long-stay psychiatric hospital Netherne in Surrey (1946–1981) and after his retirement seeing patients in his studio in West London. Throughout his professional career Adamson collected a large number of artworks produced in these sessions, the collection known today as the Adamson Collection and governed by the Adamson Collection Trust, chaired by Dr David O’Flynn from South London and Maudsley NHS Foundation Trust.

Although Adamson was instrumental in art therapy becoming a profession (he helped establish the British Association of Art Therapists in 1964), later in his life he came to consider the works in the Collection in the wider context of Outsider Art and preferred to see their creators as “artists” rather than “patients”. The Adamson Collection (around 5000 flat works) is currently deposited, but not exhibited in its entirety, in the Wellcome Library, alongside two pertinent archive collections: the personal papers of Edward Adamson (including numerous photos and slides of works) and the documents of Adamson Collection Trust.

With this paper the author wishes to consider works in the Adamson Collection from several perspectives, wondering how they were seen depending on the beholder. In the Collection’s early days (1946–1951), the works were examined to diagnose patients’ mental conditions, becoming “hard” psychiatric tools created in a scientifically controlled environment. In a more humanistic approach, influenced substantially by a discourse around surrealist art, they can be seen as a form of communication, allowing those people kept in confinement to share what mattered to them and what bothered them. Adamson’s own readings of the works, comprising of a mix of approaches, are collected primarily in his 1984 book *Art as Healing*, written collaboratively with his partner John Timlin.

## Creating art as medical experiment

Adamson held a fine art degree and his career initially developed in a commercial setting, until after the war he joined artist Adrian Hill to work with the British Red Cross Society Picture Library. The programme brought famous works of art to the patients of the Tuberculosis (TB) sanatoria, with a series of accompanying art history lectures. In 1946 Adamson visited a long-stay psychiatric hospital Netherne in Surrey, and shortly after he was offered a part time job by the medical superintendent, psychiatrist E. Cunningham Dax. Adamson became the first artist in the UK to be employed by an NHS hospital, working full time from 1948 until he retired in 1981.

As reported by medical staff, patients and the artist himself, Adamson’s rapport with the patients was of a special quality from the very beginning, as even during his first visit he was approached by people who wanted to share their creative output with him. However, between 1946 and 1951 Adamson worked as a research assistant for Dr. Dax and his colleagues, following the inflexible script of their psychiatric “experiment” involving creative activity.

Selected Netherne patients were referred to Adamson’s studio, built in 1948 from a converted army hut, to create paintings in his presence. Some of them hardly spoke or would not speak at all, so a picture was worth a thousand words indeed. Painting was seen as a therapeutic aid and not just an occupational diversion, which was made clear to patients before they took up the sessions.

In an attempt to turn an art studio into a lab, the conditions of workshops were strictly controlled: up to 20 patients who could fit in the space each had: (1) a green painted chair; (2) an easel of the same size (it does not seem they took the variety in patients’ height into account!); (3) a small table. They were given paper of standard dimensions (22’’x18’’), standard colour range of 11 poster paints and two brushes. Diagnostic worth of the images would have been compromised by any external influence, so Adamson was discouraged from knowing patients’ history (bar the most essential bits of information doctors chose to share with him) as well as from discussing ideas or works before they were executed. He did not partake in interpretation of the works either: that was the job of a panel of mental health professionals whose framework of reference was contemporary psychiatry rather than art history.

In this scenario patients were, somewhat perversely, granted a role of unconscious (and sometimes involuntary) illustrators of the content of their “unwell” minds, in the controlled environment supposed to reduce mediation (or indeed, a degree of their creative agency) to minimum. Typical psychiatric descriptions of the works, quoted in Dax’s 1953 book *Experimental studies in psychiatric art*, and in his 1948 article in *Nursing Times*, include: “Painting by a patient with an acute anxiety state. The inability to reach a firm position and her fear of the consequences of falling are dramatically portrayed in this picture”;^1^ “These are pictures typical of affective psychoses”.^1^ “The paintings of maniacal patients are, as one would expect, highly coloured”, writes Dax, “with free use of the brush, lacking in restraint, often obscene, untidy, bold, careless and dirty”.^2^

More controversially for today’s sensibilities, psychiatrists also compared the images created before and after leucotomy, then a standard procedure. The works were analyzed not only from the vantage point of the pre- and post-operation mental landscape illustrated, but also considering how the patient’s *artistic* ability was influenced: “the work…will help elucidate the effect of emotional disturbance on artistic products”.^2^ It seemed leucotomy was detrimental for patient’s creativity, with the work losing its emotional and social appeal, although a brief outburst of “creative spell” immediately after leucotomy took place in some 20% of Dax’s patients.

E. Cunningham Dax moved to Australia in 1952, taking some of the Netherne works with him. He started the Cunningham Dax Collection, now housed in Melbourne and containing over 15,000 creative works. Interviewed for *History of Psychiatry* in 1998, he expressed quite bluntly how he sees that collection:

The point of our art is that ours is a clinical collection. I don’t care two damns what the painting looks like or its artistic value or anything else. I am not interested in that, merely in the painting as an expression of the particular sort of illness.^3^

Dax catalogued works by illness, and in the same interview he fondly remembers how they had “35 different schizophrenic categories”. Although he ostensibly advised against displaying patients’ work in any way, emphasising that “the only reward is the satisfaction to the patient in its construction and in the uses to which it can be put in its treatment”,^2^ the Cunningham Dax collection was not the first time he broke his own rule. In 1950 he showed around 20 works created by Netherne patients at the ground-breaking International Exhibition on Psychopathological Art at the Sainte-Anne hospital in Paris that took place in conjunction with the first International Congress of Psychiatry. Showcasing more than 2000 works, the exhibition was seen by 10,000 people: not all of them were medical professionals and works were not viewed as diagnostic tools, “illustrations of disease”, but rather as pieces raising awareness about the world of people living with mental illness.

## Adamson’s studio after the experiment: free expression

After Dax’s departure that marked the end of “the experiment”, Adamson could put into practice his ideas, especially his belief that unimpeded creation of art is therapeutic in and of itself. When assisting Dax, Adamson found that patients were willing to “pour out” their souls on paper without much prompting so he stuck to this “non-interventionist” method of work. Over the years, he perfected his role of creating and maintaining an atmosphere where patients felt accepted and relaxed enough to follow their creative impulse. Such facilitating of creative process by passive presence and observation is akin to the “child-centred” approach to art teaching. High artistic quality of many of the works in the Collection confirms the effectiveness of this approach when combined with Adamson’s unique personal traits.

In his post-1951 work, Adamson drew not only on non-interventionism as requested by Dax, but equally on his experience from other sessions he lead at the same time as “the experiment”, working with 30 chronically ill women in a ward. These were very different from the prescribed conditions in the studio, according to Dax:

The patients paint for their own pleasure, they are not united by a common bond of working to get better, nor with a label of treatment attached…these long-standing patients show their enjoyment, revelling in the use of colour and in the boldness of their expression.^2^

The studio remained important as a therapeutic space apart from the “desert of sameness” the hospital was. Some patients now came to work with him without their doctors’ referrals and Adamson highlighted the active role they played in their own healing by doing so.

Adamson had no qualms about showing works created during these sessions, but did not see it fit to exhibit in the studio itself nor on the hospital walls (“this would have brought back echoes of the schoolroom, or suggest a degree of preferment”^3^). He designed a purpose-built gallery on the hospital grounds that opened in 1956, originally “only to selected visitors”, that “served a didactic purpose for trainee doctors and nurses….a forum where the patient’s point of view could be expressed”. Later, it was visited by 3500 visitors yearly. Moving patients’ output from closed psychiatric files onto the gallery walls was controversial for some, for example, Irene Champernowne maintained that in both cases patients had no control over what happened to their works.

**Figure 1.**
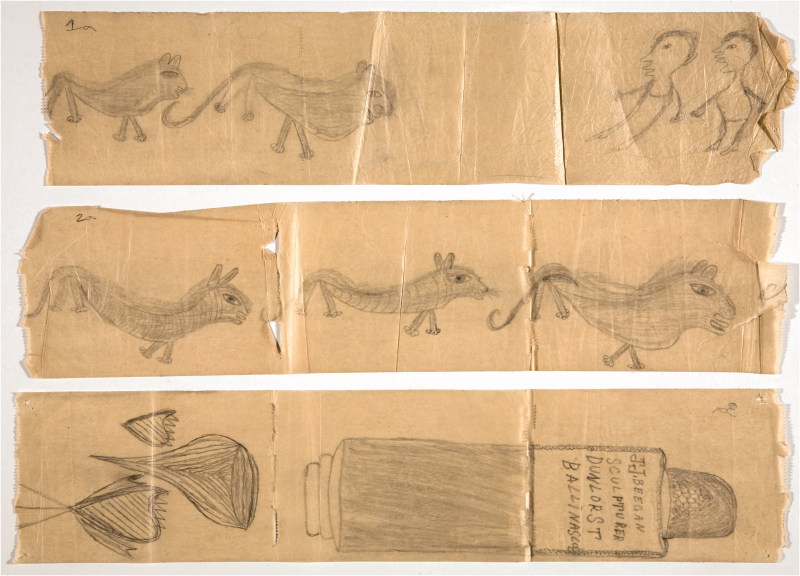
Graffiti on lavatory paper, JJ Beegan. Courtesy of the Adamson Collection Trust and the Wellcome Library.

**Figure 2.**
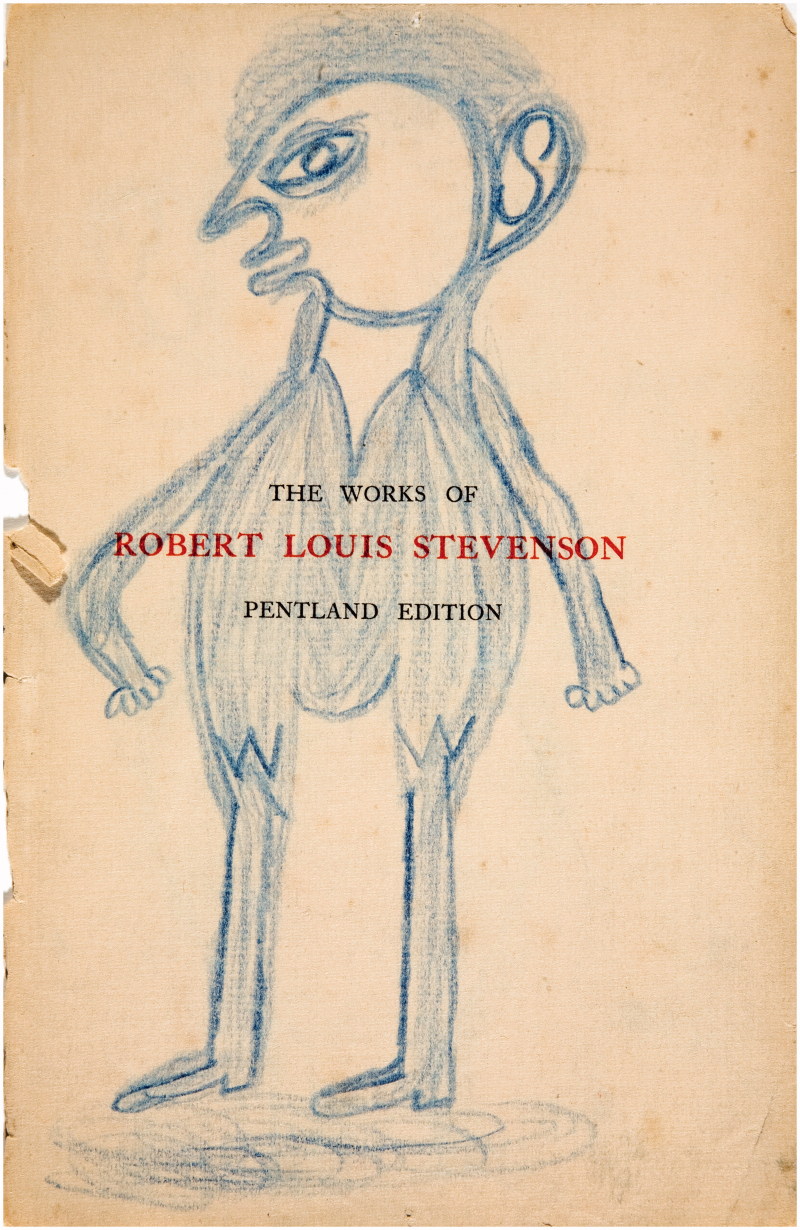
Fly-leaf drawing, JJ Beegan. Courtesy of the Adamson Collection Trust and the Wellcome Library.

**Figure 3.**
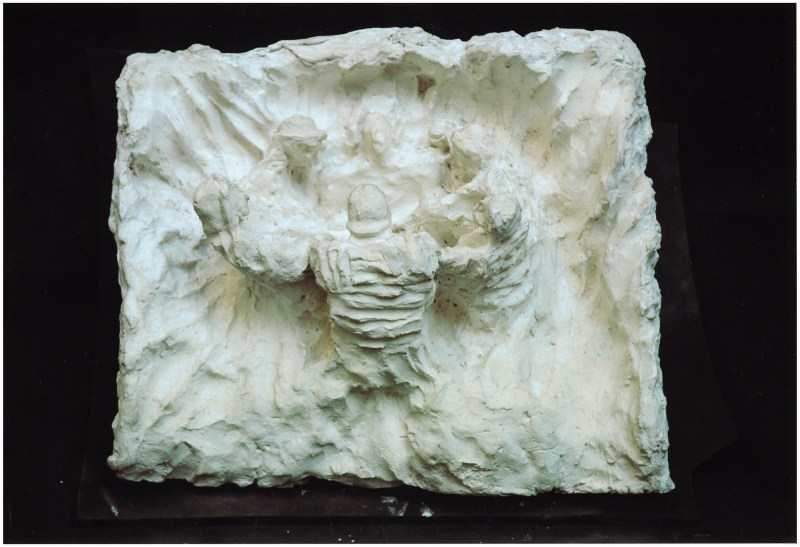
The stations of the cross, Rolanda Polonska. Courtesy of the Adamson Collection Trust and the Wellcome Library.

**Figure 4.**
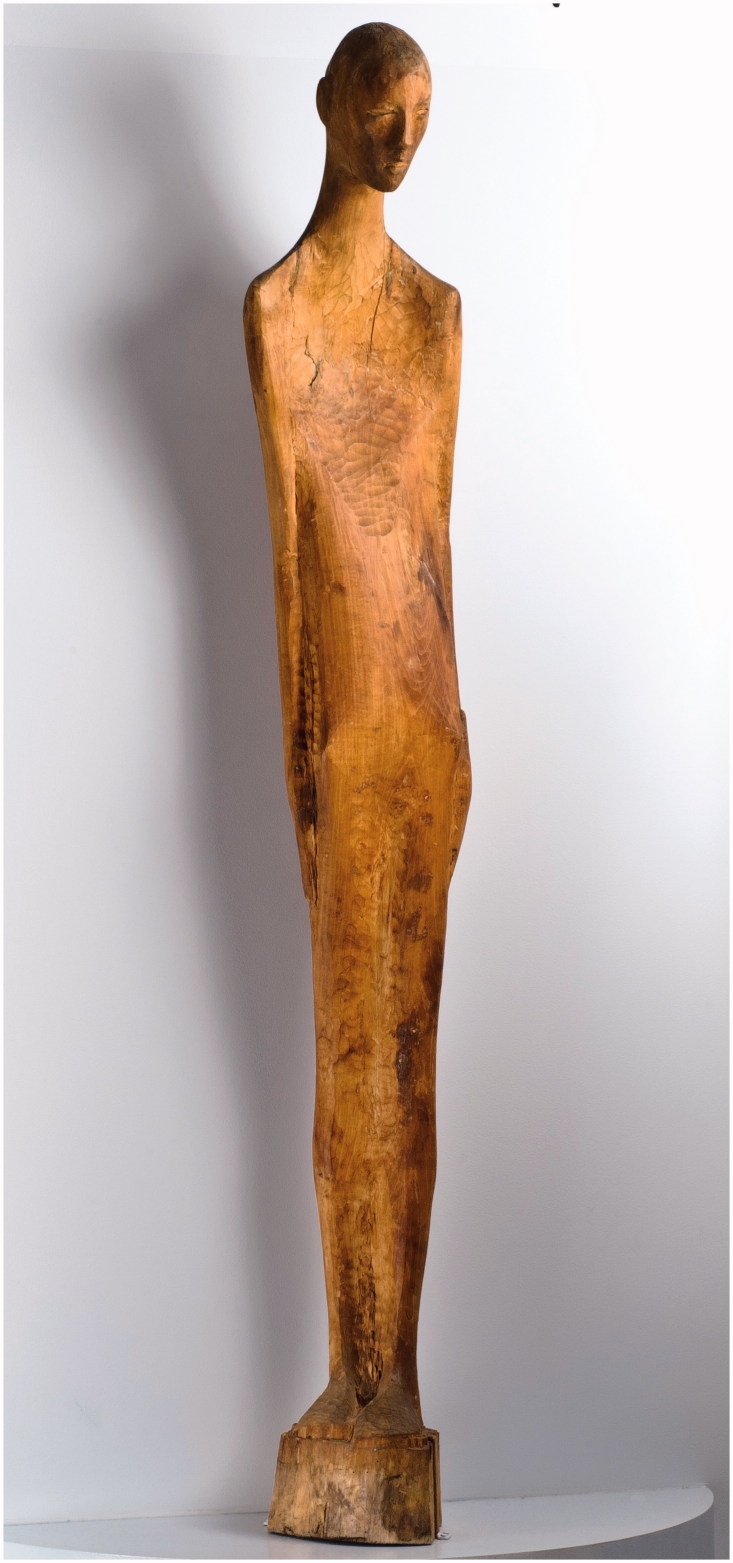
Recovery, unknown artist. Courtesy of the American Visionary Arts Museum, Baltimore.

Adamson departed from basic standardised materials, too. Perhaps inspired by his first visit to Netherne, when he was presented with works in charred matchstick on toilet paper (Figure 1) (enthusiastically received during a recent exhibition at Halle Saint Pierre gallery in Paris as newly discovered masterpieces of art brut) as well as on flyleaves torn out of the library books (Figure 2). Adamson was happy to help his artists unleash their powerful creative energy. On his watch they were using, among others, cement and wire coat hangers; the entire content of renowned engraver George Buday’s studio was transferred to “a tiny disused summer house” on hospital grounds; Gwyneth Rowlands made her unique works on found flints and pebbles. Fourteen stations of the cross by sculptor Rolanda Polonska were cast (Figure 3), which was “somewhat unpopular with the cleaners”.^4^ Adamson provided Polonska, as well as the anonymous author of “Recovery”, a large wood carving from a fallen tree (Figure 4), with tools, bypassing the hospital’s health and safety regulations.

## Art as Healing (1984)

Adamson’s *Art as Healing* features reproductions of selected works, grouped in an attempt at a certain classification. Focussing on the works themselves allows Adamson to use a wide range of interpretive frameworks at once, as if led by what he sees. While expressing his preference to “rely on the pictures to speak for themselves”, he does admit they “can be an essential key for the doctor or psychotherapist who wishes to unlock the private door into the inner world of his client’s state of mind”,^4^ which makes room for both psychoanalytical and Jungian interpretations. As some patients stayed at Netherne for 20–30 years and worked with him just as long, he also had a unique opportunity to trace their life stories and progress in the hospital in series of works. Viewed from different angles, the works are deemed to illustrate different things. Just like “mainstream” creators, Adamson artists express their feelings both in oblique, at times abstract or symbolic, way and in a straightforward, at times narrative, manner.

**Figure 5.**
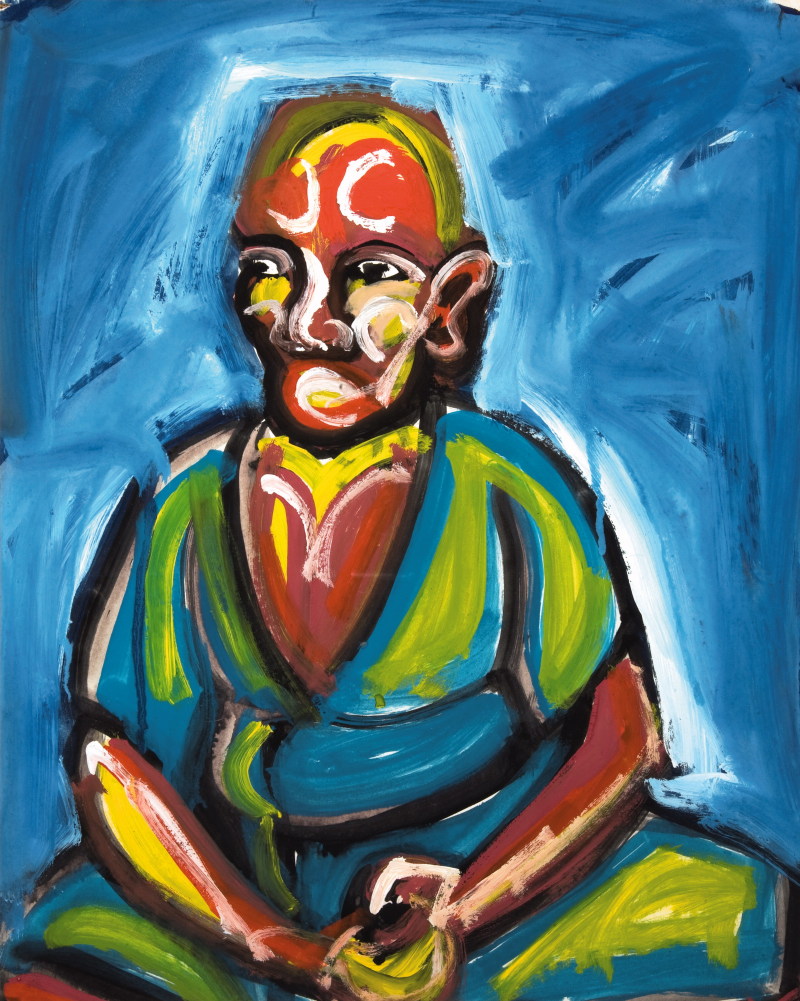
The Robed Guru, unknown artist. Courtesy of the Adamson Collection Trust and the Wellcome Library.

In some cases Adamson refers to the author’s mental condition, overlapping with a psychiatric approach, especially as he seeks the signs of impending suicide, establishing that, for example, a colour combination of red and black suddenly entering the practice augurs danger. In a chapter about the significance of dreams and how they get translated into work, the viewer is “invited to the secret garden of dreams” ^3^ and the readings do not focus on the author’s mental health but rather on the features of internal landscape we all share. Similarly, he sees some paintings explicitly not as a “further manifestation of their [the artists’] pathology”^3^ but a means of communicating with the audience, which is one of the important ways of understanding art in general. Writing about William Kurelek, a professional Canadian painter who spent several years in the UK because of his mental illness, Adamson compares the evolving content of his paintings to psychotherapy, as the artist works through the painful past experiences in his art to get healed (although Kurelek himself contested this view in his later life). In a more extreme version of such therapy, Adamson describes a series of works by an unnamed man as they parallel his life’s journey from severe depersonalisation to mental wholeness and harmony (Figure 5).

A bonus series of works — technically not part of the Collection — sees Edward Adamson himself becoming a medical illustrator of sorts. Apparently, during one of the sessions he was asked by one of the participants why he was not drawing or painting like everybody else. Thus prompted, he started sketching portraits of the attendees, now included in the Adamson’s personal archive held at the Wellcome Library.

Astonishing diversity and richness of works in the Adamson Collection call for flexibility in approaching them, both to establish their identity and to offer an interpretation. Deciding conclusively whether artistic output of people living with a range of mental conditions is an illustration of their illness or an amplified expression of their inherent creativity seems impossible: they can be seen as either or as both. In the spirit of Adamson’s humanistic approach, pieces in Adamson Collection should perhaps be seen also as touching documents of the lives of people confined to the harsh conditions of a mental asylum, thankfully one including an oasis of artistic expression in Edward Adamson’s studio.
